# Is value-based payment for healthcare feasible under Ghana’s National Health Insurance Scheme?

**DOI:** 10.1186/s12961-021-00794-y

**Published:** 2021-12-11

**Authors:** Yussif Issahaku, Andrea Thoumi, Gilbert Abotisem Abiiro, Osondu Ogbouji, Justice Nonvignon

**Affiliations:** 1 Department of Health Policy, Planning and Management, School of Public Health, University of Ghana, Legon, Ghana; 2 Fuu D/A Junior High School, Ghana Education Service, Fuu, North East Gonja, Ghana; 3 Robert J. Margolis, MD, Center for Health Policy, Duke University, 1201 Pennsylvania Ave, NW, Suite 500, Washington DC, 20004 USA; 4 Center for Policy Impact in Global Health, Duke Global Health Institute, Duke University, Durham, NC USA; 5 Department of Health Services, Policy, Planning, Management and Economics, School of Public Health, University for Development Studies, Tamale, Ghana

**Keywords:** Feasibility, Ghana, Health financing, National health insurance, Value-based payment

## Abstract

**Background:**

Effective payment mechanisms for healthcare are critical to the quality of care and the efficiency and responsiveness of health systems to meet specific population health needs. Since its inception, Ghana’s National Health Insurance Scheme (NHIS) has adopted fee-for-service, diagnostic-related groups and capitation methods, which have contributed to provider reimbursement delays, rising costs and poor quality of care rendered to the scheme’s clients. The aim of this study was to explore stakeholder perceptions of the feasibility of value-based payment (VBP) for healthcare in Ghana. Value-based payment refers to a system whereby healthcare providers are paid for the value of services rendered to patients instead of the volume of services.

**Methods:**

This study employed a cross-sectional qualitative design. National-level stakeholders were purposively selected for in-depth interviews. The participants included policy-makers (*n* = 4), implementers (*n* = 5), public health insurers (*n* = 3), public and private healthcare providers (*n* = 7) and civil society organization officers (*n* = 1). Interviews were audio-recorded and transcribed. Data analysis was performed using both deductive and inductive thematic analysis. The data were analysed using QSR NVivo 12 software.

**Results:**

Generally, participants perceived VBP to be feasible if certain supporting systems were in place and potential implementation constraints were addressed. Although the concept of VBP was widely accepted, study participants reported that efficient resource management, provider motivation incentives and community empowerment were required to align VBP to the Ghanaian context. Weak electronic information systems and underdeveloped healthcare infrastructure were seen as challenges to the integration of VBP into the Ghanaian health system. Therefore, improvement of existing systems beyond healthcare, including public education, politics, data, finance, regulation, planning, infrastructure and stakeholder attitudes towards VBP, will affect the overall feasibility of VBP in Ghana.

**Conclusion:**

Value-based payment could be a feasible policy option for the NHIS in Ghana if potential implementation challenges such as limited financial and human resources and underdeveloped health system infrastructure are addressed. Governmental support and provider capacity-building are therefore essential for VBP implementation in Ghana. Future feasibility and acceptability studies will need to consider community and patient perspectives.

## Background

Globally, increasing healthcare costs have resulted in healthcare expenditures that exceed gross domestic product (GDP) growth rates [1, 2] and often led to catastrophic spending by households. In many low-and middle-income countries (LMICs), rising healthcare costs have occurred despite low total healthcare spending measured as a percentage of GDP and as per capita spending. For example, increasing healthcare costs in Ghana are due to increases in both demand (e.g. increased coverage of the population and services provided through the National Health Insurance Scheme [NHIS]) and supply (e.g. higher cost of medical technologies) to meet population health needs. Ghana’s development over the past two decades has improved population health including increased life expectancy and reduced infant mortality [3–5]. These health improvements, along with other development factors such as increased household spending, have (1) increased the demand for health services and (2) created an epidemiological transition. The central government, healthcare providers and users, and other stakeholders in Ghana have identified rising healthcare costs as a priority health financing area to address in the country [6].

Ghana is one of a few countries in sub-Saharan Africa (SSA) that have implemented a social health insurance model, with the aim of removing cost as a barrier to quality healthcare. By increasing affordability, the introduction of the Ghana NHIS in 2004 has significantly increased healthcare utilization and reduced health disparities in access among Ghanaians in the past decade [7]. Since the implementation of the scheme, the National Health Insurance Authority (NHIA) has employed different payment models, including fee-for-service (FFS) and diagnosis-related grouping (DRG), and piloted the capitation method [8, 9]. However, these payment methods have not been able to comprehensively address the challenges of the rising cost of healthcare, delays in reimbursing providers and, more importantly, the poor quality of healthcare received by the NHIS clients [8, 9]. Problems associated with these predominant payment methods coupled with resource constraints, socioeconomic factors and lapses in health policy implementation and management have led to notably compromised quality of care that clients receive [10].

The performance of any healthcare financing system depends on the payment and care delivery mechanisms adopted. Predominant payment mechanisms that are used globally include FFS, DRG, capitation and mixed payment methods [11]. Although these payment methods have helped shape health financing in many countries, poorer health outcomes and quality of care are partly attributed to the methods by which healthcare providers are reimbursed for their services delivered [12]. For LMICs, health financing systems are one barrier to countries’ pursuit of primary healthcare (PHC) for all and attaining universal health coverage (UHC) [13]. In recent years, developed countries have been employing new payment strategies that seek to promote value for funds spent in the healthcare market, including value-based payment (VBP) models [14, 15].

Growing global interest in VBP is due to a collective call for better healthcare for individuals and populations, reduction in the cost of healthcare, and general improvement in population health [16]. VBP methods aim to improve healthcare quality while minimizing total cost by linking financial incentives to the value of health outcomes rather than volume [17]. VBP methods are designed to create new care delivery models centred on patient and population health outcomes [18]. Value is explained as improved health outcome that yields maximum health benefit at a minimum cost—through delivery processes that offer the best patient experience and minimize clinical errors [19]. Furthermore, value is conceptualized as multifaceted and encompasses not only high quality of care at the lowest possible cost, but also efficiency in cooperation, innovativeness and health promotion [20]. Yet, while many health systems have implemented VBP models as a health financing strategy, concerns about healthcare costs, efficiency, quality, affordability and equity abound [21].

VBP models vary in complexity and intended effects on quality, cost and efficiency. Models range from pay-for-performance (P4P) to bundled payments to shared savings and shared risk models [22]. In a P4P system, providers receive financial bonuses for meeting specific care quality metrics and cost targets [22]. Bonuses have not always proven to be sufficient in driving the desired change in provider practice or health outcomes [23]. Bundled payments involve fixed, predetermined fees paid to providers to perform all the associated services of a given procedure, rather than paying for each service separately [24, 25]. Thus, providers are rewarded financially for performing procedures in cost-efficient and effective ways, while avoiding unnecessary procedures and duplication of services [24]. Under the shared savings model, payers set a budget for care delivery costs as a ceiling for total costs of providers, and those providers whose total costs fall below the budget share in the savings. With the shared risk model, providers are still able to share in any recognized savings but are also expected to pay for costs that exceed the care delivery costs set by the payer [19].

Although studies have been conducted in the area of health financing in Ghana, particularly regarding enrolment and equity [26, 27], there is paucity of research exploring the use of VBP for healthcare as a channel to complement coverage improvements with care delivery models that shift the focus of care to overall population health. In the absence of a specific VBP model currently implemented in Ghana, it is important to explore VBP in its generic sense to identify the general factors that can facilitate or impede the design and implementation of a potential VBP system in the country. The Ghanaian environmental context (structure, political, legal, socioeconomic, market conditions and regulatory factors), provider capacity (financial, essential staff, infrastructure), provider and payer alignment systems, and health information systems are essential determinants of the appropriateness of VBP in Ghana [17].

While many countries have designed and implemented a wide range of specific healthcare programs that fit into VBP models, challenges in implementing any of the many VBP models vary by country context [13–15]. In Ghana, value is a channel that can encourage new care delivery models or payment for nonclinical providers (like community health workers) to extend the reach of the health system to rural or low-resource areas. In turn, VBP can serve as an alternative provider payment mechanism that Ghana considers as it seeks to extend PHC and meet UHC goals. This study therefore explored stakeholder perceptions of the feasibility of introducing the general concept of VBP as one of the alternative provider payment mechanisms that the NHIS could consider to pay for health services. Specifically, this study aimed at providing the first step towards understanding the feasibility and buy-in among policy-makers, purchasers and providers. However, given the mixed evidence of the performance of VBP models (e.g. can exacerbate inequities), the authors have not taken the strong position that VBP is superior to other provider payment mechanisms or that it is the best option for payment reform in Ghana. As the NHIS does not pay for health promotion and disease prevention, the authors did not explore VBP as a broader payment mechanism for public health interventions.

## Methods

### Conceptual framework for assessing feasibility

Bowen et al. [28] identified the following focus areas of feasibility: acceptability, demand, practicality, integration, implementation, adaption, expansion and limited-efficacy testing. These aspects are crucial in investigating the likelihood of effective implementation of an intervention. The authors noted that the outcome of a feasibility study is used to determine whether a programme, service, policy or product is appropriate for further testing. Country ownership and multi-stakeholder collaboration are vital components of any health reform effort. While VBP to date has largely been piloted in health insurance claims, there is a potential to use VBP to meet current health commitments.

The present study focused on four areas of feasibility. (1) Acceptability refers to the extent to which VBP was judged as suitable or attractive to specific stakeholders. The sample outcomes of interest included participants’ intent to support implementation, perceived appropriateness, satisfaction and perceived positive or negative effects of VBP on Ghana’s health system. (2) Practicality refers to the extent to which VBP can be carried out in Ghana using existing means, resources and circumstances without outside intervention. The effects of VBP on target stakeholders and their ability to carry out intervention activities, as well as cost analysis, were the outcomes of interest. (3) Integration refers to the extent to which VBP can be integrated within the existing Ghanaian health system. Here, we assessed the perceived fit with infrastructure, perceived sustainability, and cost to policy and implementation bodies. (4) Implementation refers to the extent to which the policy can be successfully implemented. Here, we focused on the amount and types of resources needed to implement it, factors affecting implementation, efficiency, speed or quality of implementation, degree of execution, and success or failure of execution. The assessment of feasibility based on these four areas helped to determine whether VBP is feasible in Ghana [28]. We investigated only these four focus areas in this study because VBP has never been implemented in Ghana. Thus, the demand (data on estimated or actual use of selected intervention activities in a defined intervention population or setting), expansion (the potential success of an already successful intervention with a different population or setting) and limited-efficacy testing (testing of an intervention in a limited way, with intermediate rather than final outcomes) aspects were not applicable in this study.

### Study design and sampling

A cross-sectional qualitative design was used for this study, where data collection took place in Accra over a period of 2 months. The data were collected from a purposive sample of health system stakeholders at the national level to explore their perspectives about the feasibility of VBP for healthcare under the NHIS and the perceived facilitators and barriers vis-à-vis its implementation. We purposively sampled national-level health policy-makers, NHIS staff, implementers, practitioners (physicians, midwives, nurses) and civil society organization (CSO) officers. We selected these stakeholders because of their direct knowledge and understanding of health financing, provider payment mechanisms (e.g. VBP models) and the current health financing schemes in Ghana. Hence these stakeholders were recruited to ensure data relevance. Patients and community residents were excluded from this exploratory study because VBP has never been implemented in Ghana. Therefore, these stakeholders would have less exposure to the concept of VBP and would not meet the criteria for subject matter experts within our purposive sampling criteria [29]. Resource limitations for this study was another reason for community-level exclusion. Permission and recommendation of potential participants were sought from heads of target institutions with attachments including an ethical clearance letter, consent forms and study proposal. Recommended persons were contacted by phone for further permission and arrangement of interviews. A total of 20 health system stakeholders were identified and interviewed, including policy-makers from the health ministry (*n* = 4), implementers from the GHS Head Office (*n* = 5), frontline public and private providers (*n* = 7) including medical doctors, midwives and nurses, insurers from NHIS (*n* = 3) and CSO members (*n* = 1). The criteria for inclusion were as follows: interviewees (1) understood English and (2) had worked with their institutions or similar ones for at least 3 years, to ensure that participants had some experience relevant to the study. Those who could not provide vital information to the study due to ill health or for other reasons were excluded.

### Data collection method and tool

Individual in-depth interviews (IDIs) were conducted with participants using a semi-structured interview guide that focused on the four feasibility focus areas and general facilitating and constraining factors of VBP. The data collection tool was designed by the authors based on the literature to cover the generic factors that affect VBP models including the environmental context, provider capacity, provider and payer alignment systems, and data infrastructure [17]. The data collection tool covered key areas including participants’ reaction to VBP and the extent to which it could be adopted in Ghana, how VBP fit with Ghana’s health system, and the perceived ease or difficulty in its implementation. Because of the COVID-19 pandemic, the interviews were conducted mainly by telephone and lasted between 20 and 40 minutes with each participant. Interviews were audio-recorded following participant consent. Data collection took place from August 2020 to September 2020. The data collection instrument was pretested to identify lapses, and the necessary updates were made before beginning stakeholder interviews. All data were collected by the first author, mostly by telephone interviews. The interviews ended when saturation of data was reached at *n* = 20, thus when no new information was obtained from any additional participant sampled for the study [30].

### Data analysis

All interviews were audio-recorded and transcribed verbatim into Microsoft Word 2013 by the lead author with the assistance of an experienced transcriptionist. The data were then processed and analysed using QSR NVivo 12 software. A phrase-level analytical approach was applied to all the transcripts. The transcripts were coded by two different people to identify themes under each feasibility focus area. Out of the total transcripts, four transcripts were randomly selected to be coded independently by two people to establish inter-coder trustworthiness. All initial disagreements that were identified between the two people were resolved using a consensus approach. Data analysis was performed using both deductive and inductive thematic analysis methods [31, 32]. In order to become familiar with the data, the transcripts were first read repeatedly while critically taking note of patterns within the data. Similar data types were grouped into initial categories and notes were also prepared from this process. There was a critical examination of the initial categories to ensure that each category represented participant responses before labelling these categories as codes. The themes interpreted by the researchers from the codes were used to assess the feasibility of VBP for healthcare in Ghana.

## Results

### Sociodemographic characteristics of the study participants

A total of 20 individuals participated in the study, including four policy-makers, five implementers, seven providers, three insurers and one CSO member. All the study participants had over 3 years of working experience, with the majority of participants in the 3–10-year work experience category. Table 1 provides a summary of additional sociodemographic characteristics of the study participants, including sex and age.

**Table 1:** Sociodemographic characteristics of study participants

Characteristic	Number of participants				
	Policy-makers	Implementers	Providers	Insurers	CSO
	(*n* = 4)	(*n* = 5)	(*n* = 7)	(*n* = 3)	(*n* = 1)
Sex					
Male	4	5	6	2	1
Female	0	0	1	1	0
Age (years)					
30–39	1	0	3	0	1
40–49	3	5	4	3	0
50+	0	0	0	0	0
Length of service (years)					
3–10	3	2	5	1	1
10–20	1	3	2	2	0

### Understanding of VBP

The responses from the study participants revealed that almost all participants understood VBP to mean paying for the value of service outcomes instead of paying for volumes, as explained in the literature [17, 20, 33]. Participants viewed VBP as a strategy that could ensure provider accountability for healthcare quality and costs, improve health system efficiency and effectiveness, and ensure health system responsiveness to the needs and expectations of healthcare seekers. The following quotes illustrate participants’ understanding of VBP.

Value-based payment is basically the concept where the purchaser of the healthcare, I mean, say the government, employer, consumer, and where you have the payers either public or private individuals, holds the healthcare provider accountable for both quality and cost of care.. (Implementer #2, IDI)

Patients seek healthcare and expect to derive some value from the services rendered by healthcare providers in terms of satisfaction of their health needs. And therefore within that context they must have the power to hold the healthcare providers accountable to them—that they do the right thing. Thus, providing quality services in a manner that avoids long patient waiting time and negligence and all that. So when providers are paid based on patient health outcomes or for meeting some expectations, then the payer is actually doing VBP. (Policy-maker #4, IDI)

### Perceptions of feasibility of VBP

#### Acceptability

The following subthemes were identified by participants in relation to the facilitators of and barriers to acceptability of VBP in Ghana.

#### Appropriateness of VBP

All participants considered VBP a relevant concept that needs to be discussed and explored in the Ghanaian health policy context. Participants were cognizant of the impact that the current payment methods used by the NHIA have on the quality of healthcare that providers render to clients. Participants noted that the cost and quality of healthcare in Ghana need to be addressed and that paying for value is a positive direction. Further, participants articulated the importance of increasing awareness of VBP among the public to facilitate action. The following quotes relate to the theme of appropriateness:

You can’t really extract the value of services provided under this current system. So this VBP under the NHIA would have been very appropriate and helpful. The NHIA will actually know the value of services rendered by healthcare providers and not just paying for volumes of services given to clients. So I believe that it will be of essence to try other payment mechanisms. (Policy-maker #1, IDI)

I think VBP will be useful. In fact, it is very essential in the case of Ghana’s national health insurance and that is why somehow, the NHIA has been doing that by doing evaluation at the healthcare institutions to see if things are in order before engaging providers. And that is how come sometimes when the bills are submitted, healthcare providers are “punished” by reduction from the claims they submitted. (Implementer #5, IDI)

#### Perceived impact of VBP on health system

All participants reported that VBP was an encouraging concept due to its potential advantages in the delivery of health services in Ghana. For instance, most participants from each health system stakeholder category thought that introducing VBP under the NHIS had the potential to reduce cost and waste, improve healthcare quality, increase client satisfaction and bring equity to the provision of services in the healthcare system. Participants emphasized that efficiency and effectiveness in the health system can only be achieved if VBP models are effectively implemented. The following quotes highlight how VBP would impact healthcare in Ghana if implemented:

Well,… to be honest, the current system favours the hospitals or the healthcare providers, in that if a patient comes to my hospital, anytime I do laboratory test I get money for it. Then I will do more lab test to get more money. But with the VBP, that one you are not looking at the number of test you did but rather how better is the patient at the end of the day—has the patient recovered? What is the outcome of all the services you have provided? Based on these that you are paid. I don’t know too much about the financial implication of VBP; however, it will benefit patients more than the provider depending on who bears which quantum of the financial burden. Thus, the patient might pay less premium for the insurance, because for any visit to a health facility, everything is geared toward what would make the patient better and not unnecessary services to escalate cost for the NHIA to pay. So VBP will have more benefits to patients, and I think that is the most important thing. And as a country in general, VBP will have a positive impact on our development, especially in the health sector, and it might also have some form of financial stability for the hospitals, as payment of claims will no long delay like what we are experiencing now. (Provider #5, IDI)

Another participant mentioned that VBP would have a positive impact on Ghana’s quest towards attaining an equitable healthcare system.

In my personal view, this form of payment… as I said in my earlier submission… will bring some form of equality in providing healthcare. You don’t need to be rich to get proper healthcare. You don’t need to be poor to get the lowest healthcare. Once providers are paid for value it is going to be a standardized something. So for me I believe that form of payment will bring standardization across board. (Implementer #1, IDI)

Some participants also perceived VBP as a means to sustaining the NHIS, as in the quotes below:

…if it is brought into our health system it will be very good and suitable. I believe that some of these monies paid to providers by the NHIA right now will cut down, and secondly, it will put pressure on the service providers to provide the best form of services available. It is very difficult to determine the quality of services in the current payment mechanisms. So I believe that VBP will sustain the NHIS and that will be very helpful in our health system. (Policy-maker #1, IDI)

It will be appropriate because it will lead to some sort of value for money. Because if there is a certain accountability and some transparency, the providers are likely to give what the people need. (Provider #4, IDI)

#### Participant support for VBP

When participants were asked about their intention to support VBP implementation if it is proposed in Ghana, all participants said they would support it. The participants declared their intentions to support implementation of VBP in Ghana as follows:

…yes, yes because it is good, because it sustains the scheme, it will reduce wastage, and Ghanaians will save money for a lot of things. So yes, I will support it. (Insurer #1, IDI)

I will surely support it..., you see … change, they say, is not easy. But with sensitization and education people will get to understand the importance of VBP. (Provider #6, IDI)

Personally I would support VBP, and I think that it is a reform that will restructure our insurance system. It will streamline our payment mechanisms toward bringing the very best of care to health service users. (Policy-maker #1, IDI)

If VBP is well structured, patients will definitely accept it.

One participant noted their intent to support efforts to implement VBP as long as there were no negative financial implications for patients.

Sure! I will support it because it is going to improve the quality of healthcare in Ghana. So if the government or the NHIA will be able and willing to pay providers for the desired service outcomes, why not? I will surely support it. But the possibility is also that VBP might lead to increase in premiums, and if that is going to be borne by consumers I will not support it, because in that case most citizens will not be able to subscribe with the NHIS. (Policy-maker #3, IDI)

#### Perceived acceptability challenges

Participants mentioned some factors that could limit the acceptability of VBP including political interference, resistance from healthcare providers and poor knowledge of the policy.

If prior measures are not put in place for a surety, healthcare providers will reject VBP. But for certainty government and other health insurance authorities will welcome VBP. But for providers there will be a problem unless we put effective measures prior to its implementation. (Provider #4, IDI)

So there are some other political underpinnings regarding some of these things. So partisan politics will be another thing that will have some effect on VBP implementation. Yes, so where they actually don’t have consensus on some of these issues, it becomes a challenge to implement. (Implementer #2, IDI)

Political agendas will also hinder VBP implementation. You know, Ghana, here everything is about partisan politics. Every political party will have a different view or interest with regards to this VBP method, and that could be a hindrance to this payment reform. (Policy-maker #3, IDI)

So some of the things we can look at is proper education, and getting all the actors involved to understand the VBP concept. (Implementer #2, IDI)

### Practicality

Three subthemes were identified under this theme: resource management, motivation and empowerment.

#### Resource management

With proper resource mobilization and allocation—including financial and material resources, I think Ghana should be able to implement VBP successfully without much problems. There must be adequate, regular and evenly distribution of health resources across the country. (Implementer #5, IDI)

Another thing is to have a better or adequate funding—whichever way we have to go to get a good funding to undertake that…that would be fine. Once there is funds I think VBP can be done. (Implementer #2, IDI)

Once we are going to look at outcomes and results, then it means that we are going to pay realistic tariffs and so budget or finance should be properly allocated, and all these things will come to play at the end the day. (Insurer #3, IDI)

#### Motivation

Participants indicated that paying providers based on merit, providing support for healthcare providers to be able to carry out implementation activities, and paying claims regularly were incentives for the providers to carry out their roles effectively.

The service providers are in to make money, especially the private providers…so I think that if they have actually given them a proposal that is attractive, at least something to keep them in business. But you go and do business and it’s a total lost—I think providers would not be interested because for the national health insurance a lot of people are breaking off now because government is owing them a lot of money. (Provider #2, IDI)

When a question was asked about the perceived positive or negative effects that VBP would have on the major health system actors (e.g. the government, the NHIA and those who offer health services to patients), participants indicated that VBP could reduce public sector cost and waste. Participants also noted that healthcare providers would be encouraged and motivated to offer effective and efficient services since their rewards would be based on the value of service outcomes.

As two policy-makers noted:

In the short term, it will make the government unpopular because of the inherent challenges in our health system, but it will inure to the benefit of the government in the long term because the systemic problems would have been identified and addressed to make the system function well. It will benefit the insurers because claims would be subjected to serious scrutiny in terms of quality of services provided to clients by providers. The providers will be efficient in the provision of quality services because they do not want to make losses or suffer any penalty for providing poor services. (Policy-maker #2, IDI)

I think VBP will really help health service delivery in Ghana. You know, when the service providers realize that they are paid more when they offer quality services, then their efficiency and effectiveness will become higher as compared to their current performance under the payment mechanisms we have now. (Policy-maker #3, IDI)

Similar statements were made by other participants as follows:

Generally, in my view… it will put the healthcare providers on their toes. It will serve as a check and they will have to do quality work, I mean, to merit the payment that they are going to get. And I think in that line everybody will take it serious. Once payment is based on performance and then they can be appraised based on that sort of thing, they will sit up. (Implementer #3, IDI)

It will have a great impact on our health system—a positive one of course. In terms of providers, you will get to know the serious ones—those who are ready to provide effective and effective services. (Provider #4, IDI)

However, one of the insurers had a different opinion about the likely behaviour of providers if they were going to be paid on the basis of service outcomes.

…In that case, providers will try to decrease the waste they won’t be paid for. They will find illegal practices to make sure that the wastage is still catered for, like copayment, like abstract payment for medicine or certain services that are covered. That normally happens. So when you introduce VBP, on the flip side you’re likely to get illegal practices that if you don’t control it well, can actually reduce the confidence that the people have in the scheme. (Insurer #1, IDI)

#### Empowerment

Generally, participants talked about the potential of a VBP system to increase patients’ awareness of their rights and entitlements under the NHIS. Again, some participants believed that paying for value could reduce public sector cost burden by focusing on providers of higher-quality health services, which could empower providers in terms of improving their liquidity.

Then again, the providers are now going to be on their toes because if the policy is well communicated, the people will get to know their rights and how to hold the providers accountable for the kind of services they provide. So the providers would do better, as they’re aware that this is what they must do, and if they don’t do it consumers will take them on, which affects their reimbursement. (Implementer #5, IDI)

I think the government and the insurers will get value for money. The scheme will not be overburdened. There will be money in the system to pay for healthcare when the excesses and points of wastages are abolished. And I think for the providers it will help them because, I mean, they’re in business to work and make profits. So if there is a secure, reliable system that will ensure regular source of payment, then providers will have a good cash flow to be able to operate and deliver better health services to the people. (Provider #1, IDI)

Another participant noted a productivity point of view:

So if patients are treated better as everything is geared toward quality care and not just paying for numbers, then we will have less morbidity, people will be healthy to work, and this would reflect in their productivity. Also, the general health of the population will improve. So I think VBP will have a lot of benefits. I wouldn’t hesitate to support its implementation in Ghana. (Provider #5, IDI)

### Integration

#### Data and data systems

The perception among most participants was that the data system in Ghana was not suitable for implementation of VBP.

There is still a gap in terms of data-sharing because most of our facilities still submit even health insurance claims manually. So until we all go probably electronic with all the facilities, then that better linkage can be seen, but for now it is a big challenge, a gap. (Implementer #2, IDI)

VBP will be sustainable to a higher level. However, when you look at our system in terms of technology and tools, it seems we are lacking. So, for instance, if I go to Legon hospital and I have been referred to Korle-Bu teaching hospital, there wouldn’t be any electronic sharing of my details to the referral hospital from the previous hospital visited. There should be a system where there is coordination such that anytime and anywhere you login with a patient ID number you will have access to the medical history of that patient for review. So we have to reshape our data system in order to have an effective implementation of VBP. (Provider #4, IDI)

…you know, we are in the 21st century—data management and sharing should not be a problem, but it is a problem because we have not moved to that level as a country. And so I feel that it is going to be a big challenge because we don’t have the requisite data systems. (Policy-maker #1, IDI)

You see, …there is more to be done about our data and information systems for VBP to work well. (Provider #5, IDI)

…also, we must have a strong database to help with accurate projections of the people to cover and even the funds inflow, which is currently a challenge. (Provider #1, IDI)

In contrast, other participants mentioned the strides Ghana has made in digitalization in recent years and were optimistic that data linkage and sharing would not be a significant implementation barrier. One service provider explained:

…like I said, I have never used my national health insurance before, and another thing that I was always worried about was renewal of my NHIS card. Because those days you had to go to their office and queue. It was stressful. But since the introduction of this mobile renewal system, you just have to dial a number, and once you have money in your mobile money account, you can just renew your card within seconds. Aha! So I think there will not be so much problem with data—even if there will be issues, they would be minor ones. (Provider #2, IDI)

When it comes to the data, what would have made it more qualitative may be lacking. And then, you know, the part of the world of our system, even if we go cloud, it is still going to face problems because the network system is not good. I think data infrastructure is going to be a problem. (Implementer #4, IDI)

A similar opinion was shared by another participant:

Generally, there is a movement towards this sort of cashless system, digitalization and all these things. Now, through these multitudes of identities—national identity card, health insurance, voter’s ID card… we can get a very good database to be able to implement this. (Policy-maker #4, IDI)

#### Sustainability

There were varied opinions regarding the sustainability of VBP in Ghana if implemented. Most participants were optimistic about the sustainability of VBP for health financing in Ghana as long as certain conditions were met. These conditions included reliable funding source, general acceptability of VBP, proper planning and execution of the policy, and the cost and waste reduction benefits of VBP.

Inasmuch as VBP is a good concept, okay, I think that it would be sustainable. (Implementer #2, IDI)

…so I think it will be very sustainable, but maybe in the beginning we have to do a very efficient course assessment sort of looking at ways of financing before we hit the ground to be sure that we will not run out of funds in the middle of it. (Provider #1, IDI)

I already stated that if VBP is a top priority of government, sustainability would not be a problem. (Policy-maker #1, IDI)

For VBP to be sustainable in Ghana, I think that it should be implemented in a way that it is a win–win from the very beginning for all the parties involved: the insurer, the provider and the government. With that you’d get all the parties putting up their best…to make sure that they benefit from the policy. So the providers and insurers will do their best and the government as well will provide a conducive environment for the model to flourish. (Insurer #2, IDI)

A few participants thought that VBP was not sustainable for certain reasons as cited below.

The sustainability can get to essential players in the political space and this may be a challenge. Any government that comes in and decides to pay less attention to it may be a challenge. (CSO #1, IDI)

Sustainability is the problem…that will be a challenge. As nation we are likely to fail when it comes to sustainability of policies of this kind. You know, everything revolves around financing, and as a country, that has been the major challenge for us—the ability to raise the needed capital. And even with the current health insurance system that we are operating, financing has been the major challenge. We still face problems of unpaid claims by the government or the authority involved, but VBP will require more. So sustainability will be an issue. Unless, otherwise, they have some kind of extra funding and some support externally to go that way. (Implementer #2, IDI)

#### Underdeveloped infrastructure

Participants pointed out some factors that could determine the level of integration of VBP with the existing system:

Our healthcare infrastructure is not yet developed to support the implementation of VBP in Ghana. So unless the issues of infrastructure is addressed, I think it will be a barrier. (Provider #5, IDI)

…and also, underdeveloped hospitals, for example, most of our emergency rooms in the GHS are one-room facilities for both sexes. And sometimes the same toilet is used by both sexes, they queue to go to toilet—no privacy. So you say you are paying for value, but it is not the duty of the healthcare providers to expand the facility, especially in the case of public hospitals. It is the government’s responsibility to provide those facilities in this case…. (Implementer #5, IDI)

### Implementation

Participants were asked to share their views on how they perceived the capacity of each major health system actor (e.g. providers, government and NHIA) to support VBP implementation if proposed. Participants focused more on the capacity of the service providers and the government, since both provider capacity and government policy have a direct influence on the feasibility of VBP. Most participants perceived the capacity of government to be higher and that VBP could be feasible in Ghana. Participants indicated that the government could provide human resources and equipment to improve provider capacity.

#### Government buy-in

With regards to the capacity of the major actors, let me talk on the government side only, because we are into policy-making. The NHIA people can help you with the rest. For the government side, it is the motive, you know…every move is driven by what is called a political ambition of the government. So if it is an ambition of the government, then resources can be allocated for the implementation of this payment reform. So if VBP is something that the government wants to implement, it is capable of doing it. But if it’s not part of government top priorities, then implementation will be an issue. So VBP has to be bought by the health minister and the government; then, of course, getting resources to implement it will be very simple. (Policy-maker #1, IDI)

Government is well structured and has the political willpower with them. Government is well structured to do it, but whether or not VBP will find its way onto government policy agenda is also a different issue. Because I have not heard any discourse in the public domain that will make VBP clamour to be placed on the government agenda, and that is where policy-making starts from. As for the providers, they may need to be equipped well in terms of both human resources and machines to be able to deliver quality care to clients. (Implementer #5, IDI)

#### Provider capacity-building

We have the resources to implement VBP. However, we need to create a conducive environment for the healthcare providers—that is, the health facilities, the kind of equipment they need and other supporting health workers. For that to happen, the government must also provide the resources before the service providers can also provide quality care, especially when it comes to the health insurance. (Provider #4, IDI)

…the government also has the capacity to empower healthcare providers by, let’s say, resourcing our healthcare facilities to enable them to deliver properly. Also, the NHIA has the capacity to put systems in place to monitor and track performance of service providers. And I think the providers too can do well in the area of capacity-building to be able to render quality healthcare for the people. (Insurer #2, IDI)

#### Resource constraints

Some participants thought that the major health system actors did not have the capacity to implement VBP in the context of the current Ghanaian health system. Participants shared similar views regarding the inadequacy of health professionals, financial constraints, and inadequate infrastructure and equipment in the current health system. For these reasons, participants perceived VBP as difficult to implement.

In Ghana here, I think the capacity is not that much, because when you look at the doctor–patient ratio in Ghana it is worrisome. If we had enough doctors I would say our capacity is moderate. I think right now our doctor–patient ratio in Ghana is 1:10 000+ or so. And besides, we don’t have adequate health facilities to contain the patients, so the capacity is not there. (Policy-maker #3, IDI)

…for me, I think we are not ready for VBP. You see, resourcing our healthcare facilities will be a challenge. As you are aware, majority of our hospitals are government-owned and are mostly underdeveloped or under-resourced. And even the private facilities, not all of them have what it takes to provide the kind of services that are expected in a VBP system, and don’t forget that funding is always a problem in Africa, not excluding Ghana. So I think serious work really need to be done before we can implement VBP. (Provider #5, IDI)

The lack of proper funding or adequate funding to carry out activities will actually hamper the implementation of VBP. (Implementer #2, IDI)

We need adequate funding…we have to be sure of how to make the money, how to manage it and how to allocate it. (Provider #6, IDI)

Our healthcare infrastructure is not yet developed to support the implementation of VBP in Ghana. So unless the issues of infrastructure is addressed, I think it will be a barrier. (Provider #5, IDI)

## Discussion

This was the first study to explore the feasibility of VBP as a potential alternative provider payment mechanism for the NHIS of Ghana. The study discovered many feasibility-related issues concerning the acceptability, practicality, integration and implementation of VBP in Ghana [28]. In general, VBP was perceived as feasible in terms of its conceptual relevance, practicality and acceptance by key health system stakeholders. However, we discovered, in line with others [34–36], that many supporting systems are needed, and current health system constraints would need to be addressed to fully establish the feasibility of VBP in Ghana. Our findings on the determinants, including facilitators and barriers, of VBP feasibility in Ghana are discussed below along our feasibility conceptual framework of acceptability, practicality, system integration and implementation capacity.

### Acceptance and feasibility

The acceptance of VBP and understanding of the importance of the payment method among all health system stakeholders are crucial to VBP implementation success. Acceptance of VBP determines the extent of contextual readiness for its execution as a locally driven payment reform and fosters multi-stakeholder collaboration [17]. Due to poor understanding and misconception regarding the capitation system, acceptance of this payment model was low among providers and clients in Ghana, which adversely affected its perceived implementation feasibility [8, 37]. There was a general perception of relatively low healthcare quality under the capitation method in the Ashanti region of Ghana where it was piloted [38–40]. However, participants in our study acknowledged the relevance of VBP as a potential solution to addressing the cost escalation and waste that are associated with the current FFS payment model.

Participants believed it was appropriate to introduce VBP under the NHIS, as they perceived the payment model to be compatible with Ghana’s health system goals and culture. Unlike the resistance to the capitation system by subscribers and providers, the outcome of this study indicates a higher potential support for VBP among health system stakeholders on the basis of perceived appropriateness and fit within the organizational objectives of the NHIS [28]. These positive findings of the perceived acceptability of VBP in Ghana must be viewed with caution, as potential barriers to acceptability such as political interference, resistance from providers and poor general population knowledge of the VBP concept were reported by our study and others [41, 42]. In addition, our study focused on exploring the concept of VBP among health system actors, and did not include community or patient perspectives, which would need to be explored in future research.

### Key facilitators and barriers regarding practicality, integration and implementation

A review of six case examples of VBP initiatives by Conrad et al. [17] in three different regions of the United States revealed an array of facilitators that influenced successful implementation. The facilitators included, but were not limited to, stakeholder consensus on the need to bring spending on healthcare under control; the existence of legislative, social and regulatory conditions for reforms of payment methods; robust governance and action support from provider and consumer organizations, major purchasers and health plans; and the availability of an all-payer claims database that could be linked to an electronic health record system, in addition to a favourable market. These findings corroborate the findings of Kruk [40], which identified three facilitators that could be established at both local and regional levels to support the success of value-based care models, and they include data standards (a common data system embraced by all stakeholders in a health system), capabilities (health system workforce capable of operating value-based care models) and knowledge (evidence on value-based care to guide policy-makers, providers and payers).

Although most participants perceived Ghana’s data infrastructure to be weak, participants also believed that the systems have the flexibility to accommodate the necessary changes to implement VBP. Participants identified the need to upgrade the central-level data system to improve data capture and information-sharing to facilitate the implementation of the policy. This finding matches that of a study conducted by Castaldi et al. [43], which concluded that all essential data must be in electronic form and linked in order to introduce a P4P programme. Some participants also thought that Ghana had made tremendous strides in the area of digitalization and cashless systems, which in addition to system flexibility will make the issue of weak data infrastructure easier to deal with. An improved electronic information management and healthcare infrastructure are therefore recommended for the integration of VBP and its sustainability within the Ghanaian health system.

Participants assumed that provider capacity could be intensified with government support through the provision of human resources and equipment. Specifically, most participants noted that provider capacity and willingness to carry out implementation activities had a strong bearing on the success of the payment reform. However, participants perceived the doctor–patient ratio and health equipment in Ghana as inadequate for VBP implementation. Participants noted that these barriers could be addressed with government commitment and support. For example, all participants said there should be initial required capital available before VBP can be adopted and a reliable source of funding to ensure its sustainability. A previous study conducted in Tanzania also identified these perceived challenges relating to weak infrastructure and resource availability [44].

Participants thought that VBP would lower the government’s and NHIA’s financial burden, as cost and waste would be reduced. According to participants, a lower financial burden could provide a source of financial empowerment for both payers and providers to operate and render quality healthcare to the Ghanaian population in an effective and efficient manner. Theoretically, the savings generated by reducing low-value care and waste could be redirected to increase staffing and other essential medicine and equipment for provision of quality care. These steps are critical for providers to align their operations with any value-based care model [40]. Increasing essential staffing, medicine and equipment would have a positive effect on the development of healthcare facilities and empowering providers as well. Bowen et al. [28] posit that empowerment adds to the practicality of a policy, programme or intervention, as well as enhancing stakeholders’ ability to carry out implementation activities. Participants also thought that rewards based on the value of service outcomes could encourage and motivate providers to offer quality healthcare at minimal cost. These positive effects or benefits may result in more efficient and effective allocation and use of healthcare resources [45]. These findings suggest that a supportive system of proper resource management, effective provider motivation incentives and citizen empowerment are key factors that would influence the practicality of a VBP system in Ghana.

This political will factor was validated by a report of the Economist Intelligence Unit [46] which revealed that countries that choose to restructure their health systems towards a more patient-centred, value-based model have strong political will, and policy-makers are moving in the direction of patient-centric approaches. Moreover, participants noted that health financing planners could avoid individual and institutional resistance by involving all stakeholders in the planning and execution of the policy. Participants indicated that effective systems must be put in place to regulate the activities of all individuals and institutions involved.

Participants mentioned political interference, resistance from healthcare providers, poor planning, financial constraints and inadequate infrastructure as potential barriers to VBP implantation in Ghana. This finding is corroborated by Vries et al. [47], who concluded that information asymmetry, lack of start-up funding, reluctance to accept financial accountability, mismatched incentives in healthcare facilities, lack of trust due to failed reform attempts, and worsening reputation of insurers were barriers to implementation of payment reform in general, according to the experiences of participants. Also, the Economist Intelligence Unit [46] found that resistance, fragmented systems and the limitations of current healthcare infrastructure and operations were some of the barriers that confront countries that choose to move towards a more patient-centric, value-based model. Conrad et al. [17] also indicated that deficiency in data infrastructure, regulatory barriers to risk-bearing providers, familiarity of providers with FFS payment, and competition among healthcare providers, insurers, and the government for various priorities were barriers to VBP implementation.

### Implications

This study is one of few (if any) studies assessing the feasibility of VBP as a potential alternative provider payment mechanism in Ghana. The outcome of the study suggests that the health system of Ghana is flexible and capable of supporting VBP implementation, particularly under the NHIS. However, valid concerns regarding implementation readiness will need to be considered in any future VBP model design. A VBP pilot can be carried out through government commitment and support, public education and stakeholder collaboration. An effective payment mechanism is a critical component of financing healthcare, as it significantly impacts quality of care and cost containment. This study also demonstrates the need to resource and restructure Ghana’s healthcare system towards value-based care. The study also unearths a remarkable stakeholder buy-in and willingness to support VBP implementation in Ghana.

### Limitations

As the study was conducted during lockdown in Ghana due to COVID-19, almost all the interviews were conducted via telephone. The use of telephone-based interviews was a limitation of the study, as there were technical challenges during some interviews, and the researcher did not have the opportunity to observe participants’ body language when responding to questions. This limitation does not have any impact on the quality and reliability of the data collected. Another limitation was that the perspectives of patients, the community and providers other than doctors, midwives and nurses were not included in this study. In addition, we cannot draw conclusions regarding the feasibility of a specific VBP model in Ghana, as we explored a general VBP model. Future studies should assess the feasibility of different models within the Ghanaian context and should also include the patient and community perspective. Also, all stakeholders were based in Accra. As a result, this study does not include the perspectives of health system actors from other regions and cities in Ghana.

## Conclusion

This study assessed stakeholder perspectives regarding the feasibility of VBP for health financing in Ghana. We found that there was potentially high stakeholder acceptance and support for the implementation of VBP in Ghana among health system actors. Participants opined that VBP could be integrated within the existing health financing system in Ghana if the necessary supporting systems were established and potential implementation constraints overcome. Limited financial and human resources, weak health information and data management systems, potential resistance from healthcare providers and underdeveloped health infrastructure were identified as potential factors that could impede VBP implementation in Ghana. A strong political will and commitment would be required to incrementally address these barriers in order to enhance the feasibility of VBP within the Ghanaian context.

## Abbreviations

DRG: Diagnosis-related grouping
FFS: Fee-for-service
GDP: Gross domestic product
GHS: Ghana Health Service
LMIC: Low- and middle-income countries
NHIA: National Health Insurance Authority
NHIS: National Health Insurance Scheme
P4P: Pay-for-performance
PHC: Primary healthcare
SSA: Sub-Saharan Africa
UHC: Universal health coverage
VBP: Value-based payment

## References

[1] Dieleman JL, Squires E, Bui AL, Campbell M, Chapin A, Hamavid H (2017). Factors associated with increases in US health care spending, 1996–2013. JAMA - J Am Med Assoc..

[2] Blumberg LJ, Waidmann TA, Blavin F, Roth J (2014). Trends in health care financial burdens, 2001 to 2009. Milbank Q.

[3] Abubakari SW, Bawah AA, Nettey EO, Apraku EA, Zandoh C, Amenga-Etego S (2019). Potential gains in reproductive-aged life expectancy if maternal mortality were eradicated from the Kintampo districts of Central Ghana. BMC Pregnancy Childbirth.

[4] Agbadi P, Agbaglo E, Tetteh JK, Adu C, Ameyaw EK, Nutor JJ (2021). Trends in under-five mortality rate disaggregated across five inequality dimensions in Ghana between 1993 and 2014. Public Health.

[5] Arnold S, Glushko V (2021). Cause-specific mortality rates: common trends and differences. Insurance Math Econ.

[6] Wedam EA, Sanyare FN (2017). Health care financing and sustainability: a study of current conceptual dialectics in Ghana. World Dev Perspect.

[7] Blanchet NJ, Fink G, Osei-Akoto I (2012). The effect of Ghana’s National Health Insurance Scheme on health care utilisation. Ghana Med J.

[8] Abiiro GA, Alatinga KA, Yamey G (2021). Why did Ghana’s national health insurance capitation payment model fall off the policy agenda? A regional level policy analysis. Health Policy Plan.

[9] Agyepong IA, Aryeetey GC, Nonvignon J, Asenso-Boadi F, Dzikunu H, Antwi E (2014). Advancing the application of systems thinking in health: provider payment and service supply behaviour and incentives in the Ghana National Health Insurance Scheme—a systems approach. Health Res Policy Syst.

[10] Amo HFH, Ansah-Adu K, Simpson SNY (2013). The provider payment system of the National Health Insurance Scheme in Ghana. Stud Soc Sci..

[11] De Bruin SR, Baan CA, Struijs JN (2011). Pay-for-performance in disease management: a systematic review of the literature. BMC Health Serv Res.

[12] Awoonor-williams JK, Tindana P, Dalinjong PA, Nartey H, Akazili J (2016). Does the operations of the National Health Insurance Scheme (NHIS) in Ghana align with the goals of Primary Health Care ? Perspectives of key stakeholders in northern Ghana. BMC Int Health Hum Rights.

[13] Thoumi A, Bandara S. Achieving health gains on the way to universal health coverage in Africa. 1–5. 2019. https://www.brookings.edu/blog/future-development/2019/02/01/achieving-health-gains-on-the-way-to-universal-health-coverage-in-africa/.

[14] McClellan M, Udayakumar K, Thoumi A, Gonzalez-Smith J, Kadakia K, Kurek N (2017). Improving care and lowering costs: evidence and lessons from a global analysis of accountable care reforms. Health Aff.

[15] McLellan M, Kent J, Beales S, Macdonnell M, Thoumi A, Shuttleworth B, Cohen S. Accountable care: focusing accountability on the outcomes. World Innovation Summit for Health. DOHA. December 2013.

[16] Squitieri L, Bozic KJ, Pusic AL (2017). The role of patient-reported outcome measures in value-based payment reform. Value Health.

[17] Conrad DA, Vaughn M, Grembowski D, Marcus-Smith M (2016). Implementing value-based payment reform. Med Care Res Rev.

[18] Berenson RA. Moving payment from volume to value : what role for performance measurement ? Health Policy. 2010. (Timely Analysis of Immediate Health Policy Issues 2).

[19] Conrad DA (2015). The theory of value-based payment incentives and their application to health care. Health Serv Res.

[20] Cattel D, Eijkenaar F, Schut FT (2020). Value-based provider payment: towards a theoretically preferred design. Health Econ Policy Law.

[21] Putera I (2017). Redefining health: implication for value-based healthcare reform health and outcomes are set for specific medical conditions. Cureus.

[22] Bailitt Health. Categorizing value-based payment models according to the LAN alternative payment model framework: examples of payment models by category. (February), 1–13. 2018. http://shvs.org/wp-content/uploads/2018/02/SHVS_APM-Categorization_Brief-Final.pdf.

[23] Mendelson A, Kondo K, Damberg C, Low A, Motuapuaka M, Freeman M (2017). The effects of pay-for-performance programs on health, health care use, and processes of care: a systematic review. Ann Intern Med.

[24] Hussey P, Mulcahy A, Schnyer C. Bundled payment: effects on health care spending and quality. 2012. www.ahrq.gov. 10.23970/ahrqepcerta208.1 PMC4781468 24422914

[25] Martin J. Preparing for the transition to value-based reimbursement: what you need to know. 3711. 2015. 30047701

[26] Nsiah-Boateng E, Nonvignon J, Aryeetey GC, Salari P, Tediosi F, Akweongo P, Aikins M (2019). Sociodemographic determinants of health insurance enrolment and dropout in urban district of Ghana: a cross-sectional study. Health Econ Rev.

[27] Nsiah-Boateng E, Prah Ruger J, Nonvignon J (2019). Is enrolment in the national health insurance scheme in Ghana pro-poor? Evidence from the Ghana living standards survey. BMJ Open.

[28] Bowen DJ, Kreuter M, Spring B, Cofta-Woerpel L, Linnan L, Weiner D (2009). How we design feasibility studies. Am J Prev Med.

[29] Quinn K (2015). The 8 basic payment methods in health care. Ann Int Med.

[30] Strijker D, Bosworth G, Bouter G (2020). Research methods in rural studies: qualitative, quantitative and mixed methods. J Rural Stud.

[31] Khan SN (2014). Qualitative research method: grounded theory. Int J Bus Manag.

[32] Roy S, Hansen AR, Ross L, Larson R (2018). A qualitative study to assess barber perceptions of the feasibility of the employer as a health advisor for obesity prevention. Am J Men’s Health.

[33] Chee TT, Ryan AM, Wasfy JH, Borden WB (2016). Current state of value-based purchasing programs. Circulation.

[34] Maru C, Lin A, Kagubare J, Stern A, Rao N, Walker J. Leapfrog to value. 2020. https://static1.squarespace.com/static/5db772d44638535b2114f2e6/t/5ddea52751c72e2c5edacd77/1574872363413/2019_LeapfrogToValue.pdfhttps://www.leapfrogtovalue.org/flagship-report.

[35] Miller G, Babiarz KS (2014). Pay-for-performance incentives in low- and middle-income country health programs. Encycl Health Econ.

[36] Ogundeji YK, Sheldon TA, Maynard A (2018). A reporting framework for describing and a typology for categorizing and analyzing the designs of health care pay for performance schemes. BMC Health Serv Res.

[37] Agyei-baffour P, Oppong R, Boateng D (2013). Knowledge, perceptions and expectations of capitation payment system in a health insurance setting : a repeated survey of clients and health providers in Kumasi, Ghana. BMC Public Health.

[38] Andoh-Adjei FX, Nsiah-Boateng E, Asante FA, Spaan E, Van Der Velden K (2018). Perception of quality health care delivery under capitation payment: a cross-sectional survey of health insurance subscribers and providers in Ghana. BMC Fam Pract.

[39] Andoh-Adjei FX, Spaan E, Asante FA, Mensah SA, van der Velden K (2016). A narrative synthesis of illustrative evidence on effects of capitation payment for primary care: lessons for Ghana and other low/middle-income countries. Ghana Med J.

[40] Kruk ME. Leapfrog to value. 2019. www.leapfrogtovalue.org.

[41] Carbone G (2011). Democratic demands and social policies: the politics of health reform in Ghana. J Mod Afr Stud.

[42] Grebe E (2017). The politics of social protection in Ghana: policy reform in a competitive African Democracy (2000–2014). SSRN Electron J.

[43] Castaldi S, Bodina A, Bevilacqua L, Parravicini E, Bertuzzi M, Auxilia F (2011). Payment for performance (P4P): any future in Italy?. BMC Public Health.

[44] Olafsdottir AE, Mayumana I, Mashasi I, Njau I, Mamdani M, Patouillard E (2014). Pay for performance: an analysis of the context of implementation in a pilot project in Tanzania. BMC Health Serv Res.

[45] Stabile BM, Thomson S, Allin S, Boyle S, Busse R, Chevreul K (2013). Health care cost containment strategies used in four other high-income countries hold lessons for the United States. Health Aff.

[46] The Economist Intelligence Unit. Value-based healthcare: a global assessment. 2016. https://renovatiobiomedica.com/wp-content/uploads/2017/03/EIU_Medtronic_Findings-and-Methodology.pdf.

[47] De Vries EF, Drewes HW, Struijs JN, Heijink R, Baan CA (2019). Barriers to payment reform : experiences from nine Dutch population health management sites. Health Policy.

